# Highly Selective Optical Sensor Eu (TTA)_3_ Phen Embedded in Poly Methylmethacrylate for Assessment of Total Prostate Specific Antigen Tumor Marker in Male Serum Suffering Prostate Diseases

**DOI:** 10.3389/fchem.2020.561052

**Published:** 2020-11-19

**Authors:** Mohannad Garoub, A. H. Hefny, W. E. Omer, Mostafa M. Elsaady, Mohamed M. Abo-Aly, Ali A. Sayqal, Ahmed Alharbi, Ahmed Hameed, Hussain Alessa, A. O. Youssef, Ekram H. Mohamed, Ayman A. Gouda, R. El Sheikh, M. N. Abou-Omar, Maged A. El-Kemary, M. S. Attia

**Affiliations:** ^1^Occupational Health Department, Faculty of Public Health and Health Informatics, Umm AL Qura University, Makkah, Saudi Arabia; ^2^Chemistry Department, Faculty of Science, Ain Shams University, Cairo, Egypt; ^3^Institute of Nanoscience & Nanotechnology, Kafrelsheikh University, Kafr ElSheikh, Egypt; ^4^Department of Chemistry, Faculty of Applied Science, Umm Al-Qura University, Makkah, Saudi Arabia; ^5^Department of Analytical Chemistry, Faculty of Pharmacy, The British University in Egypt (BUE), Cairo, Egypt; ^6^Chemistry Department, Faculty of Science, Zagazig University, Zagazig, Egypt; ^7^Department of Chemistry, Faculty of Women for Arts, Science and Education, Ain Shams University, Cairo, Egypt

**Keywords:** specific antigen, quenching, luminescence, optical sensor, prostate cancer

## Abstract

A low-cost, simple, and highly selective method was used for the assessment of total prostate specific antigen (tPSA) in the serum of prostate cancer patients. This method is based on quenching the intensity of luminescence displayed by the optical sensor Eu (TTA)_3_ phen/poly methylmethacrylate (PMMA) thin membrane or film upon adding different concentrations of tPSA. The luminescent optical sensor was synthesized and characterized through absorption, emission, scanning electron microscopy (SEM), and x-ray diffraction (XRD), and is tailored to present red luminescence at 614 nm upon excitation at 395 nm in water. The fabricated sensor fluorescence intensity is quenched in the presence of tPSA in aqueous media. The fluorescence resonance energy transfer (FRET) is the main mechanism by which the sensor performs. The sensor was successfully utilized to estimate tPSA in the serum of patients suffering prostate cancer in a time and cost effective way. The statistical results of the method were satisfactory with 0.0469 ng mL^−1^ as a detection limit and 0.99 as a correlation coefficient.

## Introduction

The PSA protease is manufactured by the prostatic gland cells whether normal or malignant. Its function is digesting the gel formed in seminal fluid after ejaculation (Schröder et al., 2014). In the case of prostate cancer, men exhibit elevated levels of total PSA and lower levels of the free form (fPSA). The fPSA/tPSA ratio can contribute to deciding whether the elevation in the level of PSA is caused by prostate malignancy (Partin et al., 1996). The PSA test is sensitive to prostate cancer but is not specific where false positive results may occur in other diseases as prostate benign hyperplasia, prostatitis, prostate intraepithelial neoplasia, acute urinary retention, and renal failure (Nadler et al., 1995). Owing to the suboptimal performance of the tPSA test, its significance as a sole test for the diagnosis of prostate cancer is not recommended as it may direct the suspected patient to administer drugs that may affect their quality of life or lead him to perform unnecessary invasive biopsies (Tkac et al., 2019). The role of the tPSA test could be used as a stand-alone test to detect the possible recurrence of prostate cancer, and monitor disease progression following treatment, irrespective of the treatment modality. Furthermore, tPSA can detect an early-stage of prostate cancer that would be missed by a digital rectal examination (Van der Kwast et al., 2003; Tkac et al., 2019). The tPSA normal level lies below 4.0 ng mL^−1^ (Tkac et al., 2019).

Recently, different approaches were utilized to overcome the over diagnosis of tPSA as an independent test including biomarker panels such as the prostate health index and/ or a combined platform of biomarkers and some clinical manifestations and variables such as the 4 K score, also known as 4-kallikrein, and the Stockholm 3 test (Ferro et al., 2020; Jin et al., 2020). These approaches utilized the tPSA levels as an important parameter. Thus, the development of novel analytical methods and the fabrication of cheap but yet sensitive sensors for the accurate estimation of tPSA is always in demand.

**Figure 1:**
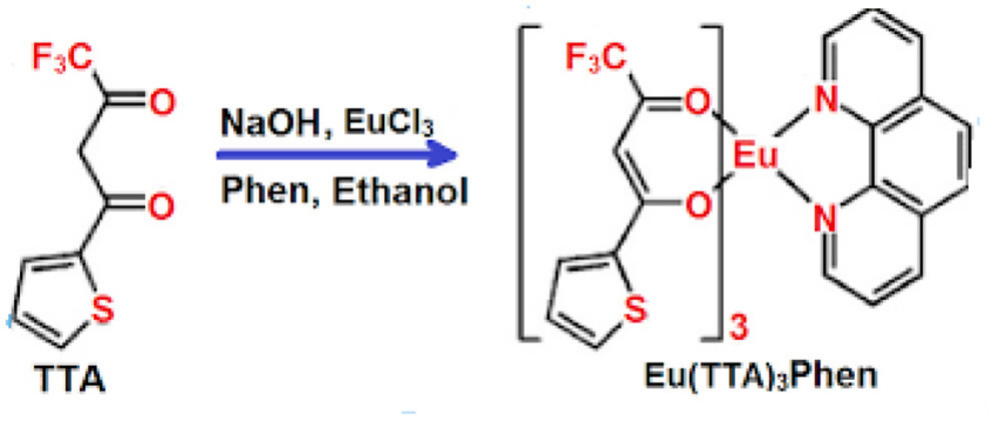
Structure of Eu (TTA)_3_ phen.

Several procedures have been described for the determination of tPSA in serum samples, such as electrochemical immunosensor (Ge et al., 2013), immunoassay (Huhtinen et al., 2004), immuno-chromatography (Yuhi et al., 2006), enhanced Raman scattering (Chen et al., 2012), surface plasmon resonance, integrated microfluidic systems (Grubisha et al., 2003), digital rectal examination, and fluorescence microscopy (Kerman et al., 2007). However, these methods have definite disadvantages where the interactions between antigen and antibody are accompanied with high constants of affinity, leading to single-use systems. Although an immunosensor is the most specific and highly sensitive method used in the laboratory (Panini et al., 2008), it is a time-consuming and expensive technique. Many recently developed methods depend on nanoscale biosensors for cancer detection at its earliest stages (Attia et al., 2019). In the present work, the optical sensor Eu (TTA)_3_ phen (Figure 1) embedded in a polymethylmethacrylate (PMMA) matrix is used for sensitive determination of tPSA as a prostate cancer marker in human serum. We determined tPSA concentration in blood serum by fluorescence quenching of this optical sensor. This is a relatively simple and inexpensive technique providing a quick reproducible analysis and is relatively free from interference with coexisting substances.

## Experimental

### Apparatus

A spectrofluorophotometer [Shimadzu RF5301PC] was used for scanning all luminescence spectra within the range of (200–800 nm). A spectrophotometer [Shimadzu UV 2450] was used for scanning the absorption spectra in 1.0 cm matched silica cells within the range of (200–800 nm). A morphology investigation was executed using SEM [Sirion, FEI] supported by an EDX detector [S-3400 N II, Japan]. Phases and crystallinity characterization of the Eu (TTA)_3_ phen nanostructure was accomplished via an x-ray diffractometer specified by Cu-kα radiation of λ =1.5412 Å, 30 mA, and 40 KV [Shimadzu 6000, Japan] the 2θ ranged between 10° and 80° with a scanning rate of 2°/min at room temperature.

### Sample Collection and Delivery

The samples of patients were supplied in agreement with WHO approved protocol for the collection of human specimens and their use in the field of research by two reputable hospitals in Egypt, Ain Shams Specialized Hospital and the New Al-Kasr-EL-Aini Teaching Hospital. The approval and consent forms of the patients to use their own samples were fulfilled before starting the experiments. The experimental clinical samples included human serum samples of different patients with normal and abnormal PSA concentrations to diagnose prostate carcinoma.

### Materials and Reagents

Uric acid, glucose, urea, albumin, KCl, and NaCl were purchased from Sigma-Aldrich. Polymethylmethacrylate (PMMA), polyethylene glycol (PEG), and tetramethoxysilane (TMOS) were purchased from Alfa-Aesar. Total prostate-specific antigen (tPSA) (1 mg) was purchased from (Ortho-Clinical Diagnostics). A system of Milli Q-Plus was used for the production of pure distilled water (Millipore Corporation, USA). Pure solvents of analytical grade were utilized throughout the whole work (Aldrich, USA).

A quantity equivalent to 53.4 ng mL^−1^ of tPSA stock solution was obtained via the dissolution of 1 mg of tPSA in 2 mL deionized water and stored at 4°C. More diluted solutions (0.1–31.5 ng mL^−1^) of tPSA were obtained through diluting the previously prepared stock solution using deionized water. The optimum temperature for saving the prepared solutions stably ranged between 0 and 4°C.

For the fabrication of the luminescent sensor, a specific amount Eu (TTA)_3_ was accurately weighed and dissolved in DMSO to prepare a stock solution with a final concentration of (5 × 10^−3^ mol L^−1^). The stock solution was further diluted by DMSO to obtain a working solution of concentration (1 × 10^−4^ mol L^−1^).

### General Procedures

#### Preparation the Optical Sensor Eu (TTA)_3_ Phen Complex Embedded in PMMA Matrix

Doping of the optical sensor (Eu (TTA)_3_ phen) in PMMA was done via adding 1.50 g PMMA to 10 mL CHCl_3_ with continuous stirring for 30 min until complete dissolution at 60 °C. Then, 200 μl of Eu (TTA)_3_ phen was added with continuous stirring for 30 min until a homogenous matrix was formed.

The solution was finally casted in a 60 × 15 mm petri dish and kept at 25°C until the solution was completely dry. The thin film thickness was 0.1 mm, and its width and height were 8.5 and 25 mm, respectively.

#### Recommended Procedure

Standard solutions of different concentrations of tPSA were accurately prepared in water. A constant definite sized Eu (TTA) _3_ Phen/PMMA film was sunk in each solution sequentially in the spectrofluorometer cell carefully to avoid its fracture. The film was rinsed with water after each measurement.

The luminescence intensity of the solutions was measured in a quartz cell of 1 cm thickness of the spectrofluorometer, at λ_ex_ = 395 nm, and the calibration graph was fitted via plotting the values of (F_0_/F – 1) at λ_em_ = 614 nm vs. tPSA concentration.

### Determination of tPSA

#### Standard Method for tPSA

##### Assay Principle

The principle procedure of the assay was reported previously, for which the PSA test was a two-site immunoenzymatic similar to a sandwich assay conducted by Kuriyama (Kuriyama et al., 1980).

In the one reaction flask, a serum sample was added to two anti-PSA monoclonal solutions, one conjugated with alkaline phosphate and the second one was used as a coat for paramagnetic nanoparticles.

A linkage between serum PSA and its anti monoclone were fixed on the solid phase together along with other interactions between the specific antigenic sites of PSA with the anti-PSA monoclone conjugate.

Magnetic separation of the solid phase from unbound materials occurred by washing. A chemiluminescent substrate, Lumi Phos—530, was dripped in the same flask leading to the generation of light that could be measured using a simple luminometer. The relationship between PSA concentrations in the sample and light generation was found to be proportional and a multi-point calibration curve was used for calculating the analyte concentrations.

##### Assay Protocol

All prepared reagents were mixed thoroughly without foaming before use. All measurements were performed in duplicate. Quantities of 25.0 μL of standards, samples, or controls were placed inside each well for a 5 min incubation period in a temperature range between 18 and 25°C. Then, 100.0 μL PSA was placed in each well-separately and merged by stirring the plates (10 s), before incubation at room temperature for 1 h (18–25°C). A quantity of 100.0 μL of solution of the TMB-substrate was added to all the wells before incubation for 20 min at 18–25°C. A quantity of 100 μL/well stop solution was added (in the same order as for the substrate solution). Absorbance (OD) was obtained at 450 nm (at 630 nm for the blank).

### Proposed Method for tPSA

For measuring the tPSA concentrations in samples of serum, the film of the optical sensor was inserted in the quartz cell of the fluorimeter then a volume of 200 μL of each was added and diluted with 1.5 mL distilled water. The intensity of the emission for the sensor was recorded at 614 nm before and after the serum was added.

## Discussion and Results

### The Absorption and Emission Spectra

The thin film of the Eu (TTA)_3_ phen /PMMA matrix in distilled water exhibited two absorption bands at 280 and 395 nm owing to π-π^*^ transitions of the organic moieties; 1, 10-phenanthroline and 2-thenoyltrifluoroacetone (Rajamouli et al., 2017a).

While upon excitation at 395 nm, the optical film exhibited six emission bands at 580, 590, 614, 650, 696, and 705 nm. These emission bands were due to excited Eu^3+^ and were characteristic to the transitions from ^5^D_0_ to ^7^F_0_, ^7^F_1_, ^7^F_2_, ^7^F_3_, and ^7^F_4_, respectively. The transfer of energy from the antenna to Eu^3+^ results in the red emission of the Eu complex in which TTA acted as a β-diketone ligand with a high absorption coefficient bound to the metal ion, while the phen ligand had an agonist shielding effect, minimized the non-radioactive rate of decay, and significantly enhanced the complex luminescence intensity (Lunstroot et al., 2010). The sensitivity of the optical sensor toward the tPSA depends on the ratio between the two emission bands at 590 nm (magnetic-dipole), where the ^5^*D*_0_ → ^7^F_1_ transition is not affected by the coordinate environment, and 614 nm (electric dipole), in which the transition ^5^*D*_0_ → ^7^F_2_ is the most intense peak, suggesting that Eu (III) occupies an inversion centered site. Any variation in the surrounding chemical environment of the sensor affects the electric dipole band (Hamed et al., 2009) of the Eu (III).

The excitation of the Eu-complex embedded in PMMA was obtained via the population of the ligands to their singlet states followed by consequent decay through the intersystem crossing (ISC) to the triplet state. The triplet state ultimately decays via a Dexter-type transfer if an antenna is found in the bonding distance field to lanthanide ion within 10 Å or less (Heine and Müller-Buschbaum, 2013). The overlap between energy levels of the antenna triplet state and the Eu(III) resonance level is an efficient triplet >5,000 cm^−1^ (Latva et al., 1997), while the most favorable difference in energy between an antenna triplet state and the Eu(III) resonance level promotes the activation of the ligand-to-metal ET route (Rajamouli et al., 2017b).

In the light of the above illustration, it may be concluded that PMMA exhibits transparent, flexible, and excellent optical properties. Hence, it could be a good host to the Eu-complex to enhance the luminescence intensity of the complex due to increasing disorder in the local environment by the surrounding polymer that decreases the symmetry of the environment around Eu ions. However, the binding of the Eu-complex to the polymer via branched groups, such as C = O and C-O, also leads to the enhanced fluorescence intensity of the Eu-complex (Zhao et al., 2006).

### Surface Characterization of Eu-Complex Embedded in PMMA

The XRD pattern for Eu (TTA)_3_ phen/PMMA film showed sharp diffraction peaks at 2⊖ = 15°, 20.6°, 21.9°, 26.6°, and 30.6° showing the formation of the crystalline structure of Eu (TTA)_3_ phen. The maximum sharp peak intensity at 45° associated with the polymer indicates that crystallinity has its maximum degree owing to the high degree of atom ordering in the polymer blends. The results suggest that the fixed crystal structure and chemical bonds were formed between the Eu-complex and the polymer matrix (Attia et al., 2012d).

While the SEM image of the as-synthesized thin film assured that the microcrystals of Eu (TTA)_3_ phen in the PMMA polymer matrix were homogenously mixed with a spherical shape, a more disordered environment, and less uniformity around Eu (III) ions of the surrounding polymer.

A demonstration of the elemental analysis of Eu (TTA)_3_ phen/PMMA thin film, which contains Eu, F, O, and C elements, due to TTA and phen, confirmed that the polymer blend and Eu-complex were synthesized successfully (Dandekar et al., 2015).

### Analytical Parameters

The emission spectra of Eu (TTA)_3_ phen/PMMA thin film in organic solvents of different polarity was examined. In an aqueous solution, the sensor displayed a high red emission intensity despite the low concentration of complexes in the matrix. The enhanced emission of Eu ions is attributed to increasing the efficiency of the ions upon excitation due to the increasing intensity of the ligand π-π^*^ transitions, which leads to the photoluminescence of Eu resulting from radiative transition from the ^5^D_0_ and ^5^D_1_ levels (Watson et al., 1975). An additional reason for increasing emission intensity in water is possibly the stabilization of electrons in the ^5^D_0_ excited state of Eu by increasing the lifetime of electrons τ^*^ = 500 μs in the excited state (Petushkov et al., 2006). Water deactivates the PL of the Eu-complex in sol gel and doping in PEG polymer techniques, as a result of the interaction with the high-frequency stretching vibrations of the hydroxyl groups (ν_OH_) that are related to the incomplete nine-coordination number of the central Eu^3+^ ion. However, in the PMMA polymer, water molecules are prohibited from Eu ions. The water molecules in the sol gel and PEG polymer techniques induce a blue shift in the fluorescence intensity of the Eu-complex due to the reduction of Eu^3+^ to Eu^2+^ in a water environment (Ugale et al., 2019).

**Figure 2:**
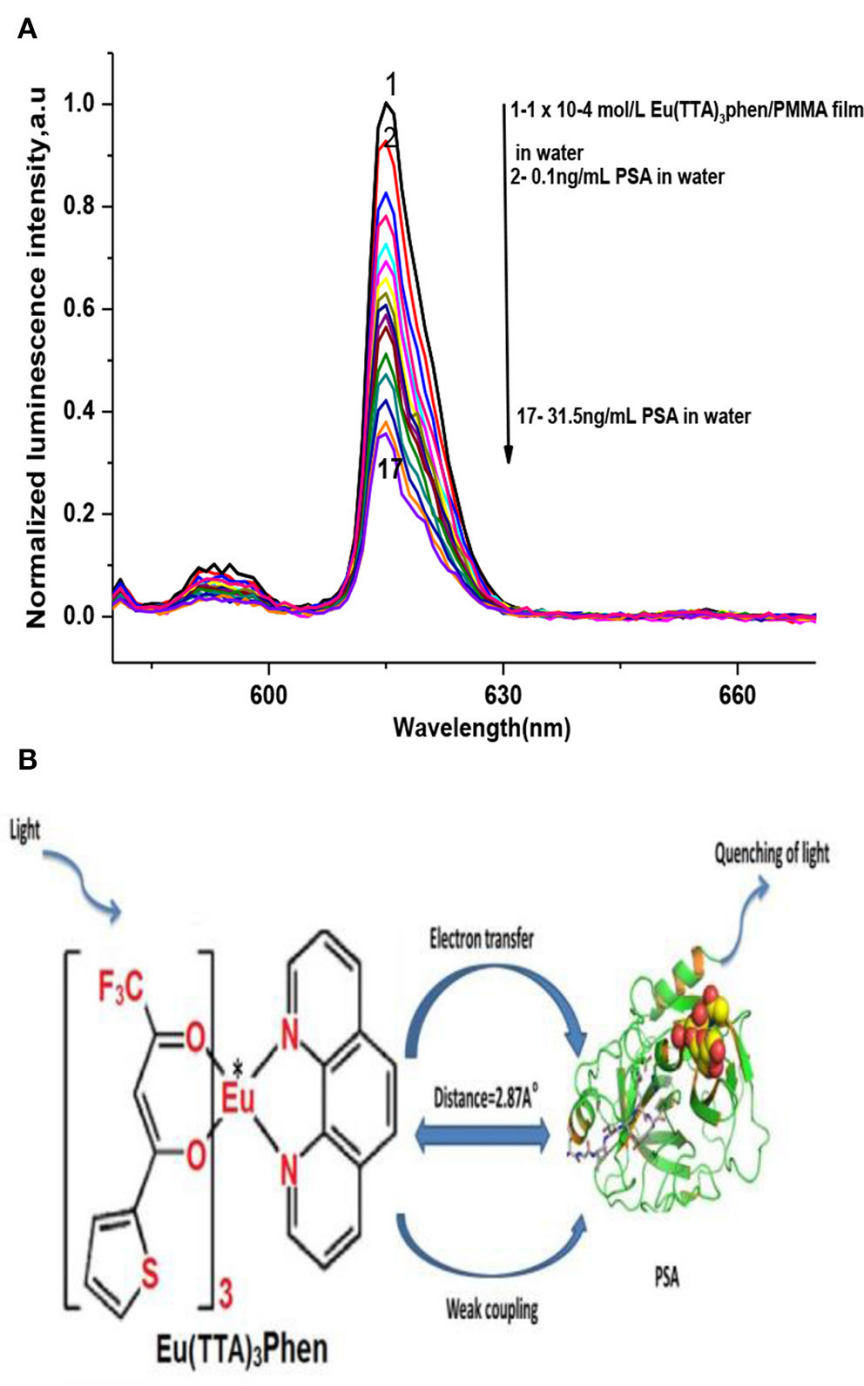
**(A)** Luminescence spectra of Eu (TTA)_3_ phen/PMMA thin film using different dilutions of PSA, at λ_ex_ = 395 nm, in water. **(B)** PSA quenching mechanism.

Figure 2A demonstrates the effect of PSA concentration levels on the luminescence power of the Eu (TTA)_3_ phen/PMMA matrix thin film under optimal experimental conditions. The luminescence intensity of the Eu (TTA)_3_ phen/ PMMA thin film was quenched by different concentrations of PSA (up to 31.5 ng mL^−1^). PSA is regarded as a quencher that interacts with the excited state of the Eu-complex by weak coupling (R = 2.87 A°), resulting in electron transfer from ^5^D_0_ to PSA by a collision mechanism, leading to the dissipation of the excitation energy of the optical sensor with less fluorescence emissions (Figure 2B).

The method selectivity and validity was investigated by studying the effect of possible interfering substances on the luminescence spectra of Eu (TTA)_3_ phen/ PMMA optical film after the addition of PSA (31 ng mL^−1^). The interfering substances included (2.0 × 10^−3^mol L^−1^) for both potassium and sodium chloride (0.06 g L^−1^), for both urea and triglycerides (0.08 g L^−1^), for glucose and uric acid (0.01 g L^−1^), total protein, and (0.7 g L^−1^) albumin. The influence of CEA, CA 15-3, and CA 19-9 was also studied in concentrations equivalent to (130 U mL^−1^) each. All of the results obtained implied insignificant influence on the sensor film luminescence intensity (Attia et al., 2006; 2012a; 2012b; 2012c; 2018; Elabd and Attia, 2016).

### Analytical Performance

**Figure 3:**
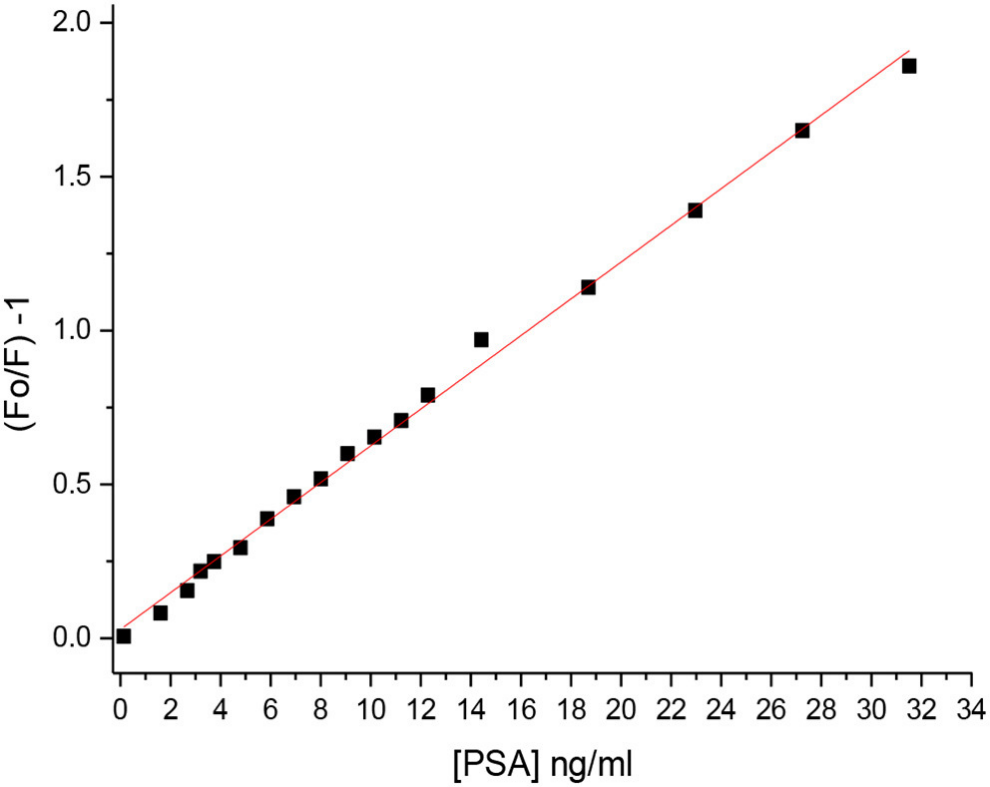
Stern–Völmer plot.

The proposed method of validation was assessed through determining the dynamic linear range, limit of detection (LOD), limit of quantification (LOQ), recovery, and precision under the improved experimental conditions (Tables 1, 2). The Stern–Völmer (SV) plot was applied for correlating the luminescence acuity of the sensor and PSA concentration in accordance with the relation; F_0_/F = 1–K_sv_ [Q] (Attia et al., 2014; 2011; 2012d; 2015; Attia and Al-Radadi, 2016a,b; 2017; Elabd and Attia, 2015; Essawy and Attia, 2013; Stern et al., 1919; Zhao et al., 2006; Dandekar et al., 2018), where F_0_ and F are the intensities of sensor luminescence in PSA absence and presence, respectively. While Q is the PSA concentration, and K_sv_ is the SV constant. A typical SV plot showed a linearity range within a concentration of (0.1–31.5 ng mL^−1^) with 0.996 as the correlation coefficient (Figure 3). SV constant is the plot slope and C_0_ = 1/K_sv_ = 16.7 ng mL^−1^. The critical radius (R^0^) was found to be 7.35/${\text{C}}_{0}^{-1/3}$ = 2.87 A.

**Table 1:** Regression parameters of the proposed luminescent method.

**Parameter**	**Values**
λ_em_ nm	614
Linearity (ng mL^−1^)	0.001–31.5
LOD (ng mL^−1^)	0.0469
LOQ (ng mL^−1^)	0.142
Intercept (a)	0.02882
Slope (b)	0.05968
Standard deviation	0.00084
Regression coefficient (r)	0.996

**Table 2:** Accuracy and precision (intra and inter-day) evaluation.

**Samples**	**Standard method average ng mL ^**−1**^**	**Intra-day accuracy and precision (*****n*** **=** **3)**					**Inter-day accuracy and precision (*****n*** **=** **3)**				
		**Average recovered ng mL** ^****−1^a^****^ **±** **CL**			**RE%**	**RSD%**	**Average recovered ng mL** ^****−1****^ **±** **CL**			**RE %**	**RSD%**
Patient (1)	8.3	8.3	±	0.001	−0.2	0.01	8.2	±	0.001	1.3	0.01
Patient (2)	3.5	3.5	±	0.002	−0.5	0.02	3.5	±	0.002	−0.2	0.02
Patient (3)	9.1	9.2	±	0.001	−0.6	0.01	9.2	±	0.001	−1.1	0.01
Patient (4)	1.2	1.2	±	0.003	0.0	0.07	1.3	±	0.003	−8.3	0.06
Patient (5)	13.7	13.7	±	0.001	0.4	0.01	13.5	±	0.001	1.4	0.01
Patient (6)	2.4	2.4	±	0.002	0.8	0.03	2.4	±	0.002	0.0	0.03
Patient (7)	6.4	6.4	±	0.001	0.2	0.01	6.3	±	0.001	0.7	0.01
Patient (8)	9.8	9.8	±	0.001	0.10	0.01	9.8	±	0.001	0.0	0.01
Patient (9)	1.3	1.3	±	0.003	−0.8	0.06	1.3	±	0.003	1.5	0.07
Patient (10)	0.9	0.9	±	0.004	3.3	0.10	1.0	±	0.004	−5.6	0.09
Patient (11)	2.7	2.7	±	0.002	−0.7	0.03	2.6	±	0.002	3.3	0.03

*CL, Confidence limits; S, standard deviation; n, number of assay*.

*The tabulated value of t is 4.303 at CL 95%*.

a *: average value = (X1+X2+X3)/3*.

The LOD and LOQ values were calculated and found to equal 0.047 and 0.142 ng ml^−1^ under established conditions, respectively. The LOD and dynamic linear range of the presented method were analog compared to the previously reported method (Wang et al., 2018). Evaluation of the method accuracy and precision (repeatability and inter-day precision) was accomplished via repeating all the assays in triplicate and all the results of the study are tabulated in Table 2. The values of relative standard deviation percentage (RSD %) were calculated to be ≤0.006–0.097% and ≤0.006–0.088% for intra and inter-day precision, respectively.

Percentage relative error (RE %) of the recovered mean concentrations and the actual PSA concentrations was measured and the results supported the high precision method. While the values of (RE %) for inter and intra-day precision were calculated to be ≤-8.33–3.33 and ≤-0.77–3.33, respectively, demonstrating that the method was highly accurate. The bias percentage was also calculated using the following equation:

[((Recovered Concentration—actual concentration) × 100)/actual concentrations]

and all the obtained results are registered in Table 2.

### Analytical Applications

For testing the applicability of the proposed luminescent method, we measured PSA concentration for 11 serum samples of patients. The results found are summarized in Table 2. It is clear that the observed data demonstrate a coincidence between the average value of PSA concentrations for patients obtained by the proposed method (13.6 ± 0.001 ng mL^−1^) and the standard method (13.7 ng mL^−1^), highlighting excellent accuracy and precision.

The adopted method is simple, time saving, and cost effective in comparison with previously reported standard chemiluminescence methods, where they usually involve time consuming and tedious manipulation steps, special expertise in the field, and sophisticated expensive instruments. Hence, the development of facile methods as an alternative for PSA estimation but yet preserving the sensitivity and specificity is of great significance. In general, the luminescent photo probe lanthanide complexes offer advantages of superior durability and fixed signal response that may extend for 24 months. Photo probes also have a constant prerogative stability over the measurements and low values of SD are obtained due to the minimization of error sources in the assays.

The limitation of the optical sensor doped in PMMA is the solubility of PMMA in nearly all the solvents except water, thus all measurements must be carried out in water at pH 7.2.

## Conclusion

The fabricated optical sensor thin film Eu (TTA)_3_ phen/PMMA matrix offers a simple and fast approach for accurate and precise determination of tPSA as an indicator for the diagnosis of prostate cancer in the early stage of the disease. The method is specific, sensitive, and cover a wide range of linearity achieved by measuring the thin film optical sensor fluorescence intensity under optimal conditions.

## Data Availability Statement

The raw data supporting the conclusions of this article will be made available by the authors, without undue reservation.

## Ethics Statement

Ethical review and approval was not required for the study on human participants in accordance with the local legislation and institutional requirements. Human samples were obtained from the New Al-Kasr-EL-Aini Teaching Hospital Cairo University and Ain Shams Specialized Hospital, Ain shams University, Cairo, Egypt in accordance with WHO (World Health Organization) approved protocol for human specimen collection and for the use of this material and related clinical information for research purposes. All patients consented and approved the using of their clinical samples in the research work. Written informed consent for participation was not required for this study in accordance with the national legislation and the institutional requirements.

## Author Contributions

All authors listed have made a substantial, direct and intellectual contribution to the work, and approved it for publication.

## Conflict of Interest

The authors declare that the research was conducted in the absence of any commercial or financial relationships that could be construed as a potential conflict of interest.
