# Supplementary material for: Highly Selective Optical Sensor Eu (TTA)3 Phen Embedded in Poly Methylmethacrylate for Assessment of Total Prostate Specific Antigen Tumor Marker in Male Serum Suffering Prostate Diseases
Source: Front Chem. 2020 Nov 19;8:561052. doi: 10.3389/fchem.2020.561052 (PMC7724366; doi:10.3389/fchem.2020.561052)
Supplement: Supplementary file 1 [file Data_Sheet_1.PDF]

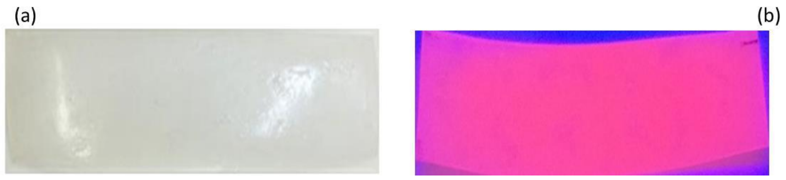

The images of Eu (TTA)3(Phen)/ poly methylmethacrylate (PMMA) film without (a) and at UV(365nm) illumination (b).

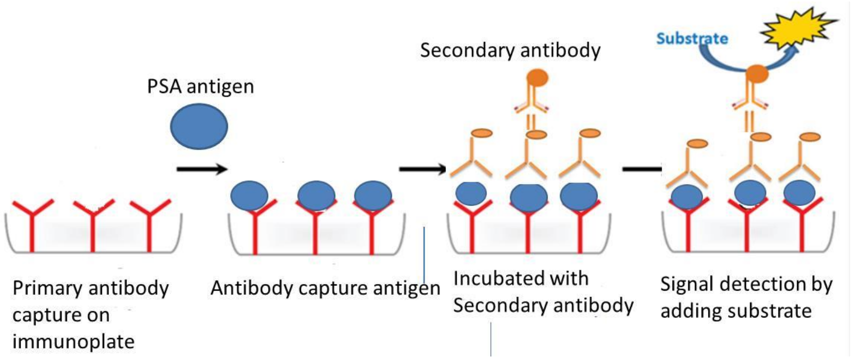

Test principle of standard method for determination of total prostate-specific antigen (tPSA).

a

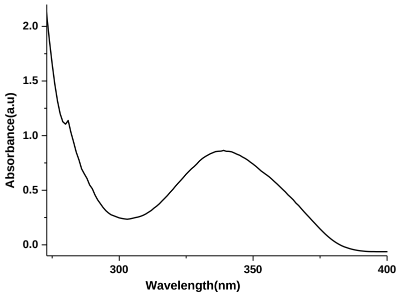

b

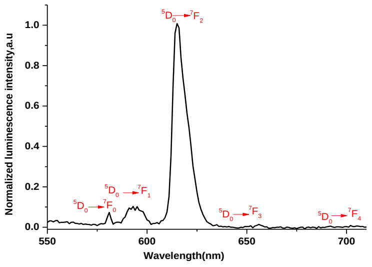

c

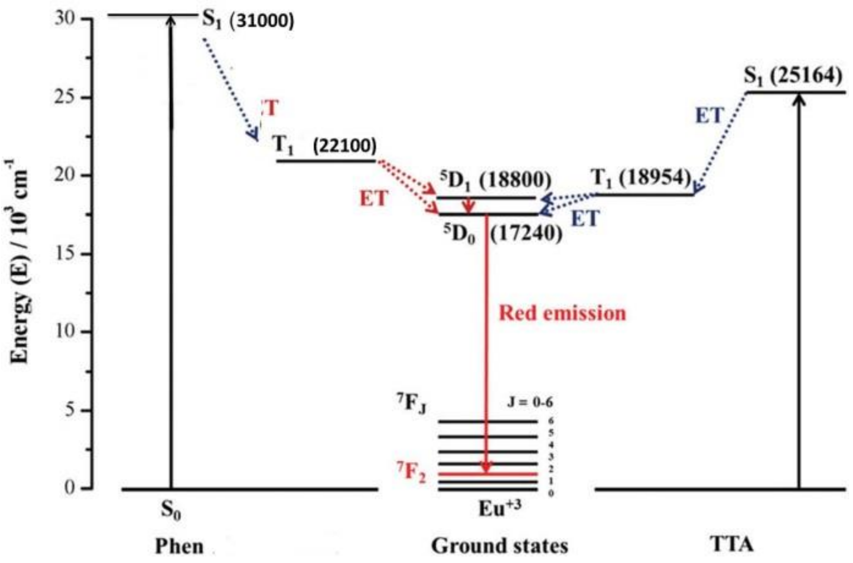

a. The UV-vis absorption spectrum of Eu (TTA)$_3$ Phen/PMMA film in distilled water.

b. Emission spectrum of Eu(TTA)$_3$ phen/PMMA Film

.The energy transfer diagram for the Eu(TTA)3Phen

(→ is non radiative)

a

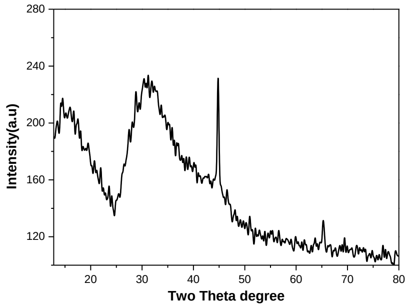

b

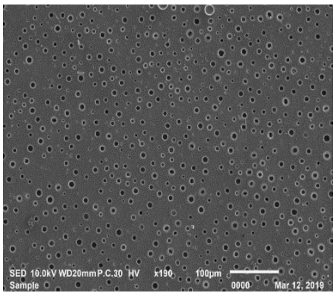

c

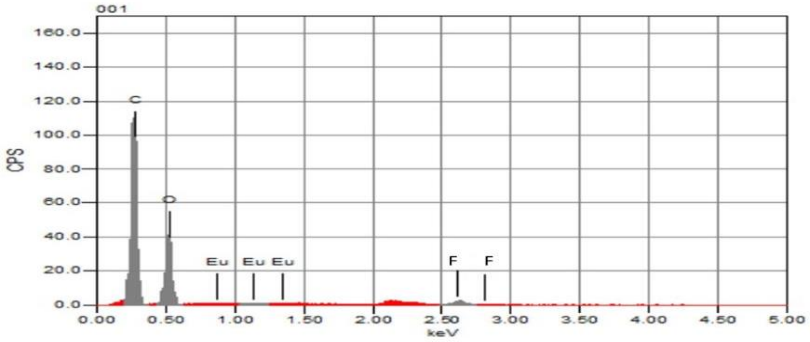

a. XRD patterns of Eu(TTA)$_3$phen/ PMMA thin film

b. SEM image of Eu (TTA)$_3$ phen/PMMA thin film.

c. EDX spectra of Eu (TTA)$_3$ phen/PMMA thin film.

<table>
<tr><td>a</td><td>b</td></tr>
<tr><td>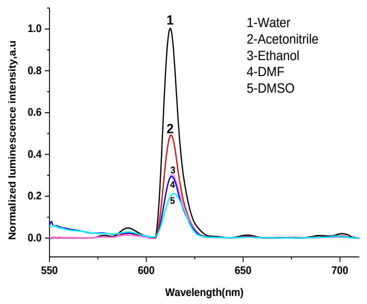
</td><td>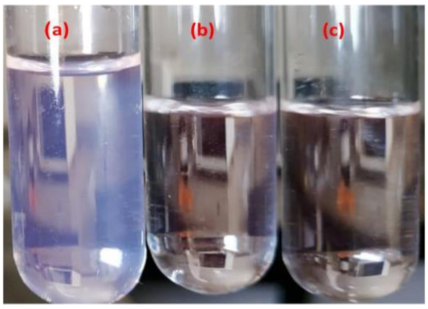
</td></tr>
<tr><td>a. Luminescence emission spectra of the optical sensor Eu (TTA)$_3$phen/PMMA thin film in different solvents.</td><td>b. Eu(TTA)$_3$phen in (a) water solution, (b) DMSO solution, and (c) DMF solution.</td></tr>
</table>
